# A novel antibacterial resin-based root canal sealer modified by Dimethylaminododecyl Methacrylate

**DOI:** 10.1038/s41598-019-47032-8

**Published:** 2019-07-23

**Authors:** Dan Liu, Xian Peng, Suping Wang, Qi Han, Bolei Li, Xinxuan Zhou, Biao Ren, Hockin H. K. Xu, Michael D. Weir, Mingyun Li, Xuedong Zhou, Lei Cheng

**Affiliations:** 1 State Key Laboratory of Oral Diseases & National Clinical Research Center for Oral Diseases, West China Hospital of Stomatology Sichuan University, Chengdu, 610041 China; 2 Department of Cariology and Endodontics, West China Hospital of Stomatology Sichuan University, Chengdu, 610041 China; 3 Department of Oral Pathology, West China Hospital of Stomatology Sichuan University, Chengdu, 610041 China; 4 Stomatological Hospital Affiliate to Zunyi Medical University, Zunyi Medical University, Zunyi, 563000 China; 5 Department of Endodontics, Periodontics and Prosthodontics, University of Maryland Dental School, Baltimore, MD21201 USA

**Keywords:** Antimicrobials, Antimicrobials, Pulpitis, Pulpitis

## Abstract

Persistent apical periodontitis, mainly caused by microorganisms infections, represents a critical challenge for endodontists. Dimethylaminododecyl methacrylate (DMADDM) is a well-studied and potent antibacterial agent used in various studies described in the literature. The aim of this study is to develop a novel antibacterial root canal sealer by incorporating DMADDM into EndoREZ and investigate the properties of the resulting material. Different mass fractions (0, 1.25%, 2.5%, and 5%) of DMADDM were incorporated into EndoREZ and the cytotoxicity, apical sealing ability and solubility of the resulting material were evaluated. Furthermore, a direct contact test, determination of colony-forming units, a crystal violet assay, scanning electronic microscopy and live/dead bacteria staining were performed to evaluate the antibacterial effect of the sealer to multispecies bacteria (*Enterococcus faecalis*, *Streptococcus gordonii*, *Actinomyces naeslundii*, and *Lactobacillus acidophilus*), in planktonic cells or biofilms. Fluorescence *in situ* hybridization and quantitative real-time polymerase chain reaction were carried out to assess the composition of the multispecies biofilms. No difference on the cytotoxicity, apical sealing ability and solubility between sealers containing DMADDM (1.25%, 2.5%) and EndoREZ (0%) could be determined. However, when the mass fraction of DMADDM increased to 5%, significantly different properties were found compared to the 0% (*p* < 0.05) group. Moreover, incorporating DMADDM into the sealer could greatly improve the antibacterial properties of EndoREZ. In addition, the composition ratio of *E. faecalis* could be decreased in multispecies microecology in sealers containing DMADDM. Therefore, a EndoREZ sealer material containing DMADDM could be considered useful in clinical applications for preventing and treating persistent apical periodontitis.

## Introduction

Persistent infections with microorganisms in the root canal represent the chief culprit to persistent apical periodontitis and endodontic failure^1,2^. However, the effective prevention and treatment for persistent apical periodontitis can not only rely on the development of new devices and technologies like microscopic root canal therapy, apical microsurgery^3^ or intentional replantation^4^, but further requires improving root filling materials, including root canal sealers and gutta percha. Root canal sealers play key roles in filling the space between core materials, or between core materials and root canal walls, or the space which core materials cannot reach, including lateral canals, apical ramification, furcation, etc^5^. Root canal sealers, with constant and effective antibacterial ability, have been shown to improve the success rate of endodontic treatments. Sealers exhibiting long-acting and potent antibacterial abilities can inhibit the growth of microorganisms, as well as reduce the occurrence of persisting bacterial after root canal filling. However, previous studies indicated that most of root canal sealers featured at least some antibacterial activity when freshly prepared, and the antibacterial ability was lost as the materials set^6^. Various research groups have tried to add some antibacterial agents such as antibiotics^7,8^, nanomaterials^7,9,10^, quaternary ammonium salts^10,11^ into root canal sealer. But most of these studies only focused on one bacteria species, namely *Enterococcus faecalis*. Generally appreciated is the notion that chronic periapical disease or persistent periapical disease is caused by multispecies bacterial infections^12^. In addition, adding traditional antibiotics such as amoxicillin to sealers trigger general concerns of drug resistance. Meanwhile, it is often questionable whether the antibacterial agents added can react with the sealer and exhibit long-time potent antibacterial abilities. Last but not least, biocompatibility of the antibacterial-modified sealer is not mentioned in most of these studies found in the literature.

EndoREZ, the second generation of a methacrylate resin-based sealer, is a hydrophilic dual-cure urethane dimethacrylate (UDMA)-based material. It can flow into accessory canals and dentinal tubules to facilitate resin tag formation for retention and sealing after smear layer removal^13^. Nevertheless, the antibacterial ability of EndoREZ is quite weak^14^. Dimethylaminododecyl methacrylate (DMADDM) represents a tape of quaternary ammonium salts (QAS) and has been studied as an antibacterial agent^15,16^. In previous studies, it was shown that antibacterial QAS monomers can copolymerize with other monomers to form polymer matrices that can reduce bacterial growth^17–19^. Both chemical structures of the main components in EndoREZ and DMADDM feature double bonds that theoretically may form cross-link structures under certain conditions. Therefore, in this study, we intended to modify EndoREZ with DMADDM and investigate the sealer biocompatibility, relevant physicochemical properties, antibacterial efficiency and influence on the composition of multispecies- *Enterococcus faecalis*, *Streptococcus gordonii*, *Actinomyces naeslundii*, and *Lactobacillus acidophilus*.

## Results

### Cytotoxicity test and physical sealer properties

**Figure 1:**
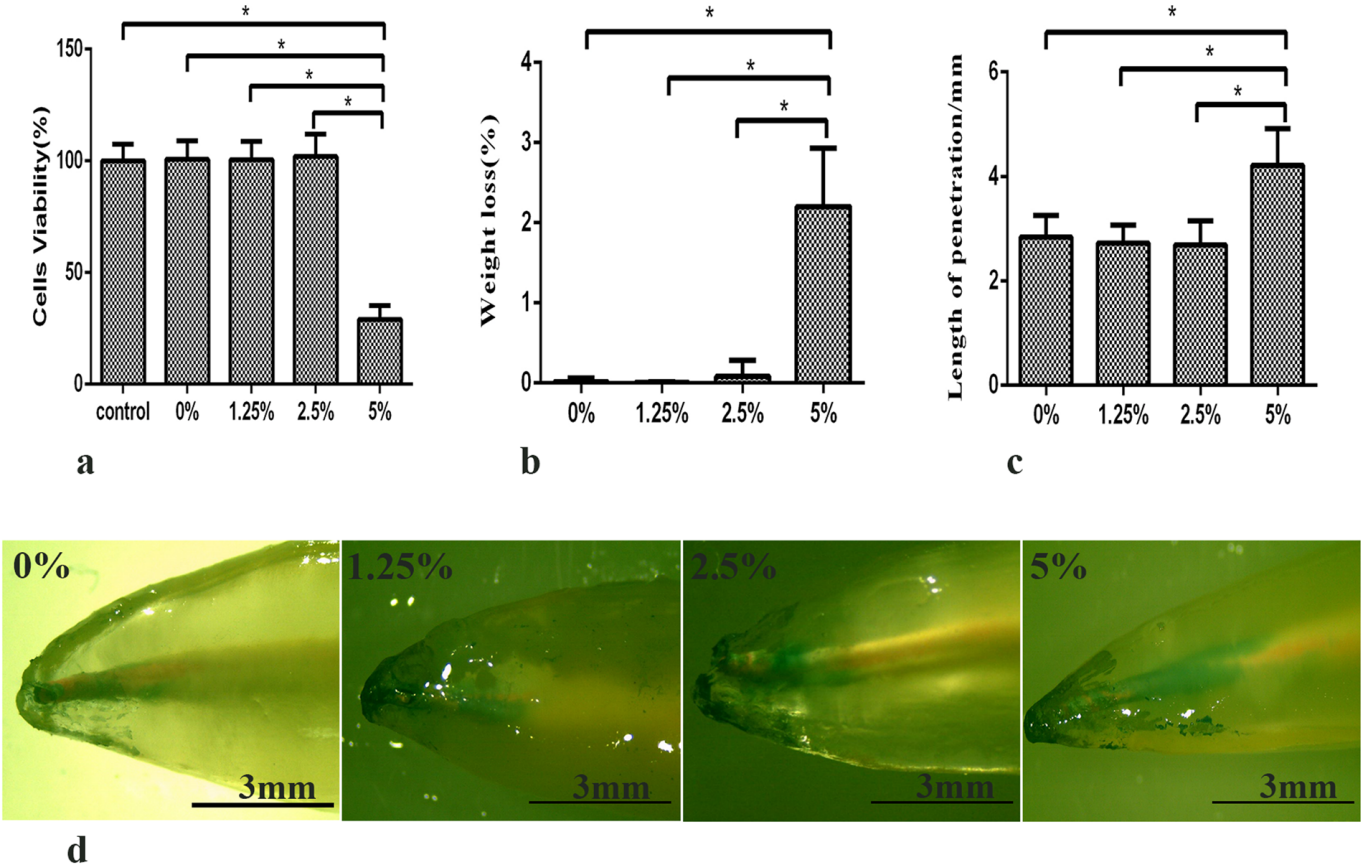
Correlation property tests of the EndoREZ containing different mass fractions of DMADDM (0%, 1.25%, 2.5%, 5%). (**a**) Cytotoxicity assay of sealer eluents with mouse fibroblast. The control group contained pure culture medium. Each values is shown as mean ± SD (*n* = 6) (*p < 0.05); (**b**) Solubility test of sealers in different groups. Each values is shown as mean ± SD (*n* = 6) (*p < 0.05); (**c**) Apical sealing ability test of the sealers. Each values is shown as mean ± SD (*n* = 6) (*p < 0.05); (**d**) Images of apical sealing ability recorded using a stereomicroscope.

The results of the cytotoxicity test are shown in Fig. 1a. It was found that the EndoREZ was non-toxic to Mouse fibroblasts (L929). Moreover, adding DMADDM at a mass fraction of 1.25% or 2.5% did not increase cytotoxicity of the sealer. However, when the mass fraction of DMADDM increased to 5%, the sealer cytotoxicity to L929 significantly increased. The solubility of the sealers in different group is shown in Fig. 1b. Though the samples were dried at 37 °C for 14 days or 30 days, the weight of the samples in the groups containing 0%, 1.25%, or 2.5% of DMADDM did not decrease even after immersion for 14 days. However, the solubility of the sealer in the group containing 5% of DMADDM was significantly increased compared to the other groups (*p* < 0.05). There was no significant difference for the depth of dye penetration (Fig. 1c,d) between EndoREZ and the sealers containing DMADDM at a mass fraction of 1.25% or 2.5% (*p* > 0.1). However, the apical sealing ability of the group containing 5% of DMADDM was significantly weaker than that of other groups (*p* < 0.05). Therefore, according to the sealer biocompatibility results, including solubility and apical sealing ability, we included in our following experiments: 0% DMADDM group, 1.25% DMADDM group and 2.5% DMADDM group.

### Antibacterial efficiency to planktonic bacteria

**Figure 2:**
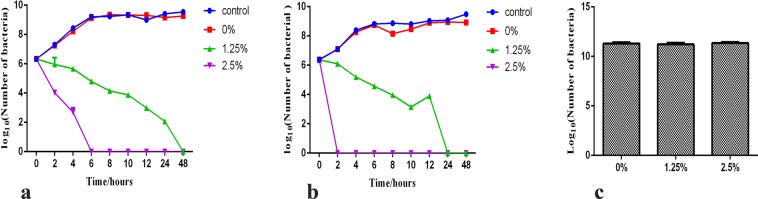
Antibacterial ability assays of the sealers containing different mass fractions of DMADDM (0%, 1.25%, 2.5%) after addition to multispecies planktonic bacteria. (**a**) Antibacterial ability of freshly prepared sealers. The control group consisted of primary bacteria suspension without any sealers. Each values is shown as mean ± SD (*n* = 4); (**b**) Antibacterial activity of the sealers set for 10 days and the control group consisting of pure bacteria suspension. Each values is shown as mean ± SD (*n* = 4); (**c**) Antibacterial efficiency of the sealer eluents, set for 10 days, added to multispecies after contacting for 24 h and 48 h. Each values is shown as mean ± SD (*n* = 6) (*p < 0.05).

The results of the direct contact test are presented in Fig. 2a,b. Inspection of Fig. 2a shows that there was no antibacterial effect of the EndoREZ (0% DMADDM group). Conversely, the sealers containing 1.25% and 2.5% of DMADDM presented a constant and effective antibacterial ability. Even after the sealers set for 10 days (Fig. 2b), no reduction of the antibacterial efficiency of the sealers containing DMADDM was found, demonstrating that addition of DMADDM to EndoREZ could add a long-time antibacterial ability to the sealer material. No antibacterial ability of the sealers eluent was seen (*p* > 0.1) (Fig. 2c), indirectly indicating that the monomer of DMADDM did not dissolve after mixing with EndoREZ.

### Antibacterial effect on multispecies biofilms

**Figure 3:**
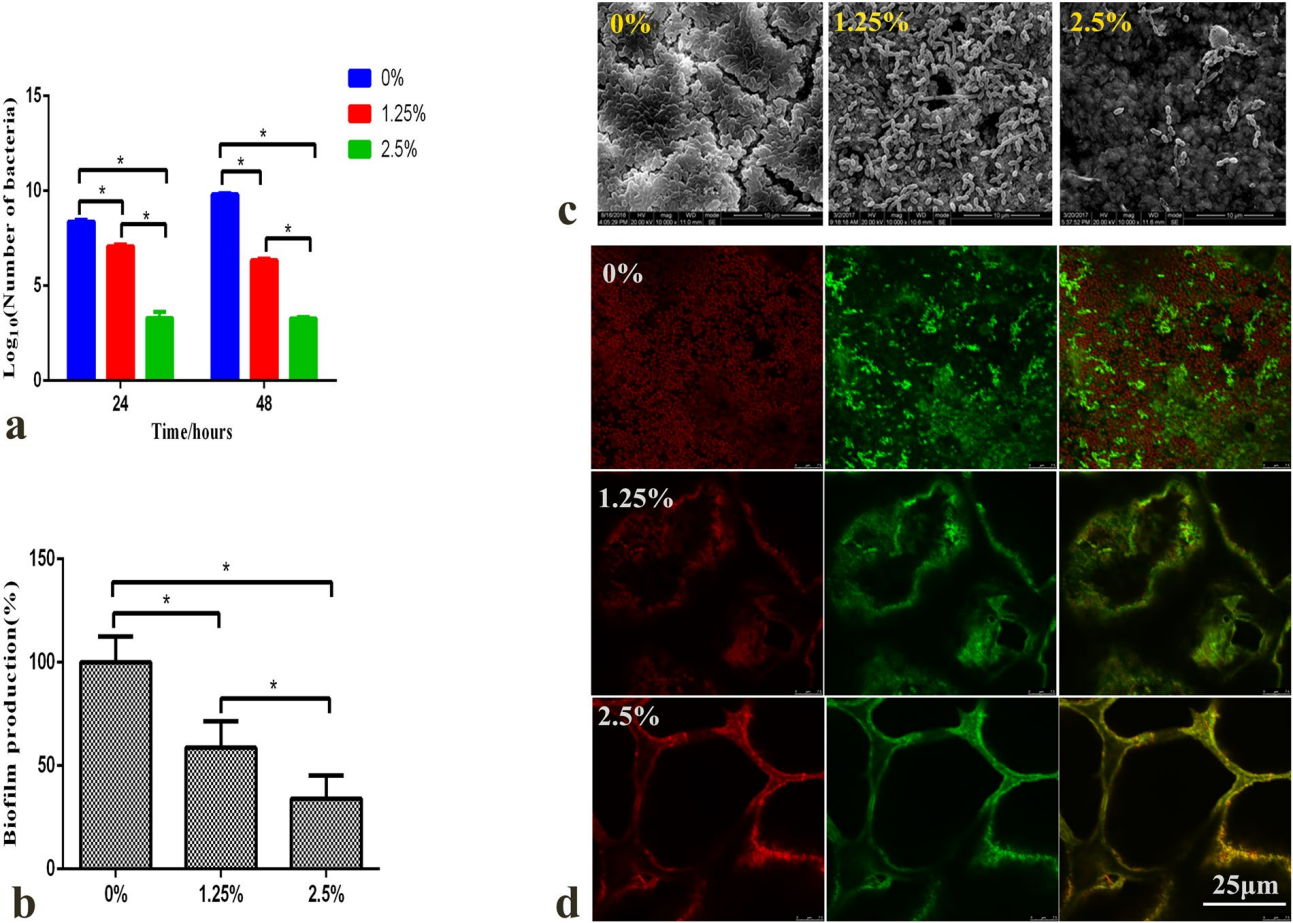
Antibacterial effect of the sealers containing different mass fractions of DMADDM (0%, 1.25%, 2.5%) after addition to multispecies biofilms. (**a**) Colony-forming unit counts of biofilms formed on each disk after 24 h and 48 h from 3 groups containing 0%, 1.25%, and 2.5% DMADDM. Each values is shown as mean ± SD (*n* = 6) (*p < 0.05); (**b**) Biofilms formation on different groups after 48 h, tested via crystal violet assay. Each values is shown as mean ± SD (*n* = 6) (*p < 0.05); (**c**) Scanning electron microscopy (SEM) images of multispecies biofilms; (**d**) Images of multispecies biofilms (live bacteria - stained green; dead cells - stained red) in different groups.

Colony-forming units (CFU) (Fig. 3a) counting in the 1.25%, 2.5% DMADDM groups were an order of magnitude less than that in the 0% DMADDM group (*p* < 0.05).The biomass of the biofilms (Fig. 3b) in the 1.25% and 2.5% groups were found to be reduced compared to that of the control group (0% DMADDM) (*p* < 0.05), demonstrating that the addition of DMADDM to EndoREZ did inhibit the biofilm formation on the surface of the sealer. The scanning electronic microscope (SEM) images in different groups are shown in Fig. 3c. Meanwhile, the biofilm of the 0% DMADDM group was much denser than the biofilm of the other two groups (1.25% and 2.5% of DMADDM). Figure 3d shows live/dead staining confocal images of biofilms on sealers after 48 h. Virtually full coverage of live bacteria could be determined in the EndoREZ group. However, the biofilms were found to be reduced in the 1.25% and 2.5% DMADDM groups compared to the EndoREZ group.

### Composition ratio of bacteria in multispecies biofilm

**Figure 4:**
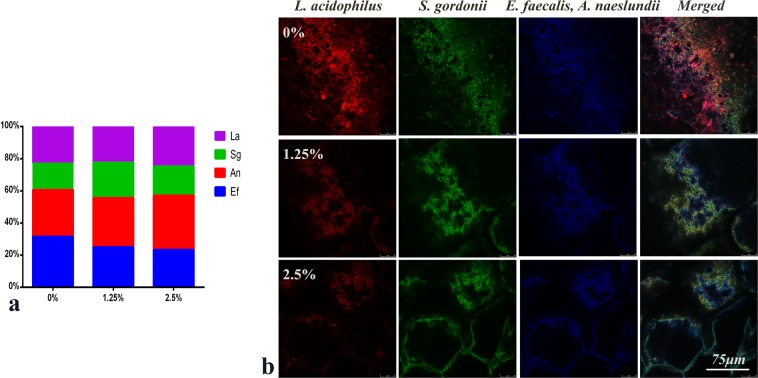
Ratios of four bacteria species in multispecies biofilms formed on the sealers containing different mass fractions of DMADDM (0%, 1.25%, 2.5%). (**a**) Ratios of *E. faecalis*, *S. gordonii*, *A. naeslundii*,and *L. acidophilus* in multispecies biofilms, subjected to TaqMan real-time polymerase chain reaction; (**b**) Fluorescent *in situ* hybridization images of multispecies biofilms (*S. gordonii* - stained green; *L. acidophilus* - stained red; *E. faecalis, A. naeslundii -* stained blue). *L. acidophilus* (red) also gave a positive signal for *E. faecalis*, however, the latter could be more clearly identified by violet color staining.

The ratio variation of the bacteria in multispecies biofilm affected by EndoREZ with different concentrations of DMADDM is shown in Fig. 4a. In the 0% DMADDM group, the proportion of *E. faecalis* reached a value of 31.42% and decreased continuously in all DMADDM groups, i.e. 24.85% and 23.26% respectively. The proportions of the other three bacteria species demonstrated slight increases. The result of fluorescence *in situ* hybridization is shown in Fig. 4b. *S. gordonii* was stained green, *A. naeslundii and E. faecalis* were stained blue, *L. acidophilus* was stained red, the probe of *L. acidophilus* (red) also showed a positive signal for *E. faecalis*. Because of this single mismatch within the binding site of this probe in *E. faecalis*, the simultaneous application of a second probe allowed for the identification of *E. faecalis* via a violet color. This combination of the probes was used to distinguish *L. acidophilus* and *E. faecalis* in the mixed community. The ratio of *E. faecalis* in the DMADDM groups was obviously lower than the ratio in the EndoREZ group.

## Discussion

Microbes are considered to play the main etiological role of endodontic disease formation. The antibacterial activity of root canal sealers could help eliminate residual microorganisms. These novel antibacterial materials may be useful complementary to chemo-mechanical preparations and intracanal medication during root canal therapy. DMADDM, a quaternary ammonium monomer, was synthesized and demonstrated to be a strong antibacterial agent in various studies reported in the literature^20–23^. In the present study, we chose DMADDM as an antibacterial agent to modify the EndoREZ sealer and investigated the resulting antibacterial effect. The result of the direct contact test indicated that EndoREZ sealer alone exhibited no antibacterial effect on the multispecies biofilms consisting of *E. faecalis, S. gordonii, A. naeslundii*, and *L. acidophilus*. Our results were consistent with previously reported studies^24,25^, which showed that this methacrylate resin–based sealer had no antibacterial activity against *E. faecalis*. Conversely, the sealers containing both 1.5% and 2.5% mass fractions of DMADDM could significantly decrease the growth of the listed multispecies, no matter of the condition of the planktonic bacteria or biofilms. In addition, the antibacterial ability of the sealers containing DMADDM was not compromised, even after setting. Materials that feature some antimicrobial properties might gradually lose their volume, thus impairing the overall quality of the seal. However, the results of the solubility tests and antibacterial ability of the sealer eluent showed that the DMADDM antibacterial-modified sealers did not lose their volume and further exhibited an effective antibacterial behavior.

In root canal infections, bacteriological studies using various techniques have identified a selected number of bacteria that persist even after root canal treatment. These bacteria include mainly gram-positive facultative anaerobic bacteria, including enterococci, streptococci, lactobacilli, and actinomyces^26^. However, most previous studies about endodontic antibacterial efficiency of sealers mainly focused on one single species bacteria, namely *E. faecalis*^27,28^. In fact, persistent periapical diseases are often caused by multispecies bacteria. In a previous study, researchers have demonstrated that *A. naeslundii*, *L. salivarius*, *S. gordonii*, and *E. faecalis* can form stable and reproducible biofilm communities^26^. Besides, some studies indicated that *E. faecalis* was more resistant to starvation when found in coexistence with *Candida albicans*, *S. gordonii*, *A. viscosus*, or *L. acidophilus*^12^. Therefore, in our present study, we selected the species *E. faecalis, S. gordonii, A. naeslundii, and L. acidophilus* to form a multispecies and investigated the antibacterial efficiency of the sealer materials.

*E. faecalis* is the most frequently detected bacteria species in root canal–treated teeth. The species has been shown to be able to adapt to harsh environmental changes, such as an extreme alkaline pH, salt concentrations, deprivation of nutrition, antimicrobial resistance, and grows in the root canal as a biofilm^12,29^. Although *E. faecalis* can be frequently found in root-filled teeth with apical periodontitis, in most cases, the strains were not isolated as a mono-infection. However, the ratio of *E. faecalis* in multispecies biofilms is considered an important index to evaluate the risk of apical periodontitis. Therefore, in the present study, a quantitative real-time polymerase chain reaction was performed to determine whether DMADDM could influence the bacterial ratio of biofilms. In doing so, we found that the sealers containing DMADDM could regulate the composition ratio of the multispecies biofilm and the presence of *E. faecalis* in the multispecies biofilms deceased upon increasing of the mass fractions of DMADDM in the sealer.

We have aslo tested the cytotoxicity of the sealer materials to ensure biocompatibility. Moreover, we have have investigated apical sealing ability to ensure that the addition of DMADDM to EndoREZ did not influence the sealers apical sealing ability. Finally, we have studied the solubility of the sealers to ensure that the quaternary ammonium monomer could not be released after mixing with EndoREZ. The results of the sealer eluent cytotoxicity, apical sealing ability and solubility were all similar. Adding DMADDM at a mass fraction of 5% had a significant influence on the sealer cytotoxicity, apical sealing ability and solubility compared with the other groups containing less DMADDM. We hypothesized that when the mass fraction of DMADDM was increased to 5%, a portion of DMADDM reacted with EndoREZ with the excess DMADDM portion still remaining anreacted. Hence, when the samples of group 5% DADDM group were immersed in cell culture medium or distilled water, free DMADDM could leak out. This may be the reason for the corresponding cytotoxicity results of the sealer eluent, the apical sealing ability and solubility of the 5% DMADDM group had a significant effect in contrast with other groups.

Using scanning electron microscopy (SEM) and LIVE/DEAD staining, we have observed the growth of biofilms on the surface of the sealers. In doing so, we have detected the distribution of the four bacteria species on the surface of different groups of sealers by fluorescence *in situ* hybridization (FISH). All images of the sealers containing DMADDM were different from that of the EndoREZ group, even in the group of sealers containing no bacteria (see Appendix). The amount of microorganisms on the surface of the experimental group was much less than that in the control group (0% DMADDM). Furthermore, a structure-like grid on the surface of the sealers containing DMADDM could be seen, however, not in the group containing 0% DMADDM. One explanation could be the formation of cross-links as a result of chemical reactions between DMADDM and EndoREZ. However, further studies are needed to confirm this hypothesis.

In conclusion, upon incorporating DMADDM (1.25% or 2.5%) to EndoREZ, a greatly improved long-term antibacterial ability of EndoREZ without compromising the biocompatibility, apical sealing ability and solubility of the sealers could be observed. Potentially, the incorporation of DMADDM could be an alternative to antibacterial sealers, ultimately improving periapical healing in endodontic treatments.

## Materials and Methods

### Ethical considerations

The study was conducted in accordance with the Declaration of Helsinki, the policy of Sichuan University and West China School of Stomatology. The protocol was approved by the Ethical Committee of West China School of Stomatology, Sichuan University (Chengdu, China) (Project identification code: WCHSIRB-D-2017-063, approval date: 02/03/2017). Written informed consent was obtained from each participant upon acquiring samples.

### Bacteria species

*Enterococcus faecalis* ATCC29212, *Streptococcus gordonii* ATCC35105, *Actinomyces naeslundii* ATCC12104, and *Lactobacillus acidophilus* ATCC4356 were provided by the State Key Laboratory of Oral Diseases (Sichuan University, Chengdu, China). The bacteria were cultured in brain-heart infusion broth (BHI; Difco, Sparks, MD, USA), anaerobically (90% N_2_, 5% CO_2_, 5% H_2_) at 37 °C.

### Synthesis of DMADDM and specimen preparation

DMADDM was synthesized and verified according to a method described previously^30^. DMADDM was mixed with EndoREZ (Ultradent, south Jordan, UT, USA), at a DMADDM (EndoREZ+DMADDM) mass fraction of 0%, 1.25%, 2.5% and 5%, respectively.

### Cytotoxicity of sealer eluent to mouse fibroblasts (L929)

The biocompatibility of the sealer was determined through testing the cytotoxicity of the sealers eluent to mouse fibroblasts. Sealers containing different mass fractions of DMADDM (0%, 1.25%, 2.5%, 5%) were injected to nonreactive plastic rings (5 mm in diameter and 2.5 mm in height), with a glass slide at the bottom using a 2-mL syringe. The samples were completely set for 10 days at 100% humidity and at 37 °C. Then, the samples were sterilized in an ethylene oxide sterilizer (Anprolene AN 74i, Andersen, Haw River, NC, Germany) and immersed in 10 mL Dulbecco’s Modified Eagle Medium (DMEM), 2% fetal bovine serum, 100 IU/mL penicillin, and 100 IU/mL streptomycin^31^. The samples were then agitated for 24 h at 37 °C to obtain the sealer eluents.

The cell line of mouse fibroblasts (L929) was cultured with 5% CO_2_ at 37 °C. The cell Counting Kit-8 (DOjinDO, Shanghai, China) was used to assess the cytotoxicity of the eluent. 4000 cells/well were placed in 96-well plates (Costar, Corning Inc., Corning, NY, USA) and incubated for 24 h at 37 °C in 5% CO_2_ atmosphere. Then, the culture medium was discarded and replaced with 100 µL of the sealers eluent. The cells were then cultured for another 24 h. After removal of the culture medium, 100 µL of fresh culture medium and 10 µL of etrazolium-8-[2-(2-methoxy-4-nitrophenyl)-3-(4-nitrophenyl)-5-(2,4-disulfophenyl)-2H-tetrazolium] monosodium salt (CCK-8 solution) were added to each well. After incubation for another hour, the solution absorbance was measured at 450 nm using a Thermo Scientific Multiskan GO reader (Thermo Fisher Scientific Inc., Waltham, MA, USA) reader. The medium without eluent culturing L929 was used as control and each group contained 6 samples.

### Apical sealing ability

Combined were the method of dye leakage with a tooth-clearing technique to evaluate the sealing property of the sealer^32,33^. Twenty four single rooted human anterior teeth with mature apex were selected from patients suffering from severe periodontitis or undergoing orthodontic treatment and the informed consent of the donors was obtained using a protocol approved by the West China Hospital of Stomatology, in Sichuan University. Routine access openings were prepared and then the working length was visually determined by subtracting 1 mm from the length of a size 10 K-file at the apical foramen. All of the canals were instrumented to a working length with a size 40 K-file using a step-back technique. The middle and coronal thirds were prepared using size 1–3 Gates-Glidden burs with a low-speed handpiece. The root canals were irrigated with 1 mL of 5.25% NaOCl while instrumenting the canals. The patency of the apical foramen was confirmed with a size 10–15 K-files. Following root canal preparation, the canals were irrigated with 10 mL of 17% ethylenediaminetetraacetic acid (EDTA) for 1 min and 10 mL of 5.25% NaOCl. A syringe with a 23-gauge needle was used for irrigation. Finally, the root canals were flushed with 3 mL of distilled water and then dried with sterile paper points. The prepared teeth were randomly divided into 4 groups of 6 teeth each. The technique of lateral condensed gutta-percha using with sealers of different DMADDM groups was used to obturate the prepared root canals. Briefly, the canal walls were coated slightly with sealer using a size 35 K-file and counter-clockwise motion. The apical part of the master gutta-percha cone was coated with the sealer from different DMADDM groups and placed into the canal. The root canals were filled with accessory cones by lateral condensation technique. The access openings were closed with light-cure flowable resin (3 M, Germany) and all specimens were kept in 100% humidity at 37 °C for 7 days to allow for the sealer to set. The root surfaces of all specimens were prepared as reported previously^34^. Briefly, the root surfaces of all specimens were coated with two layers of nail varnish and sticky wax with the exception of the apical 1 mm specimen. The specimens were then immersed in 2% methylene blue solution for another 7 days at 37 °C. After seven days, the specimens were washed with tap water and dried, and the nail varnish and sticky wax were removed with a scalpel. The samples were then demineralized in 5% nitric acid for 72 h and the solution was changed every 24 h. The specimens were rinsed with running tap water for 2 h, dehydrated in ascending concentrations of 80% ethanol for 12 h, 90% and 100% ethanol for 24 h and finally cleared and stored in methyl salicylate. Linear dye penetration was determined using a stereomicroscope (Leica, Wetzlar, Germany) with electronic Vernier caliper. All procedures were performed by the same researcher.

### Evaluation of solubility

Weight loss of the specimens was recorded as the sealer solubility^35,36^. The samples were prepared using the same method as in the cytotoxicity experiment described above. Each 10-day set sample was weighed three times in each group (n = 6) and recorded as W1. The samples were immersed in tubes containing 10 mL of distilled water for 14 days and then incubated for another 14 days at 37 °C to dry the samples. Each sample was weighed 3 times and the weight was recorded as W2. The solubility (S) was calculated using the following formula: S = (W1 − W2)/W1 × 100%.

### Direct contact test

The antibacterial efficiency of the sealer was evaluated by means of a direct contact test described previously in the literature^37^. 20 mg of the sealers were placed into 48-well plates (Costar, Corning Inc., Corning, NY, USA) using a 2-mL syringe. The fresh samples and 10-day set samples were prepared and sterilized using an ethylene oxide sterilizer. Then, 1 mL mixed inoculum containing *E. faecalis* (1 × 10^6^ colony-forming units [CFUs]/mL), *S. gordonii* (1 × 10^6^ CFUs/mL), *A. naeslundii* (1 × 10^6^ CFUs/mL), and *L. acidophilus* (1 × 10^6^ CFUs/mL), was added to each well. The well without any sealer served as negative control. The plates were incubated at 37 °C anaerobically (90% N_2_, 5% CO_2_, 5% H_2_). After cultured duration for 0 h, 2 h, 4 h, 6 h, 8 h, 10 h, 12 h, 24 h and 48 h, BHI agar plates were used to assess the microorganism viability after serial dilution in phosphate-buffered saline (PBS).

### Antibacterial efficiency of the sealers eluent

In order to determine the antibacterial efficiency of the sealers eluent, 20 mg of the sealers were placed on 48-well plates using a 2-mL syringe. The samples were then set for 10 days at 100% humidity and sterilized using an ethylene oxide sterilizer. Then, 1 mL of brain heart infusion (BHI) broth was added to each well and the 48-well plates were agitated for 24 h at 37 °C to obtain the sealers eluent. Subsequently, 500 µL of the sealers eluent was transferred to a new 48-well plate and each well contained 500 µL of mixed bacterial inoculum, containing a defined microbial population consisting of *E. faecalis* (1 × 10^6^ CFUs/mL), *S. gordonii*(1 × 10^6^ CFUs/mL)*, A. naeslundii*(1 × 10^6^ CFUs/mL), and *L. acidophilus*(1 × 10^6^ CFUs/mL). Next, the 48-well plates were incubated in an anaerobic chamber for 48 h. BHI agar plates were used to assess the microorganism viability after serial dilution in PBS.

### Fabrication of biofilm specimens

The specimens for the biofilm experiments were prepared following a study described previously in the literature^38^. Briefly, composite disks were fabricated using the cover of a sterile 48-well plate as a mold. 20 mg of the sealers containing DMADDM or the control sealer were applied on the surface of each composite disk and flatted using a spatula. The specimens were then placed into a 24-well plate and incubated at 37 °C with 100% humidity for 10 days. Next, the samples were sterilized in an ethylene oxide sterilizer. For multispecies biofilm formation, bacterial suspensions were mixed to obtain an inoculum containing a defined microbial population consisting of *E. faecalis* (1 × 10^6^ CFUs/mL), *S. gordonii*(1 × 10^6^ CFUs/mL)*, A. naeslundii*(1 × 10^6^ CFUs/mL), and *L. acidophilus*(1 × 10^6^ CFUs/mL) in 2 mL of BHI with 1% sucrose for 24 h, and 48 h at 37 °C anaerobically to form biofilms.

### Colony-forming units (CFU)

After cultured for 24 h and 48 h, the biofilms were gently rinsed twice with phosphate buffered saline (PBS) to remove the planktonic bacteria. The biofilms in each group were then harvested by scraping and sonication in PBS buffer. After preparation of serial dilutions in PBS, the bacteria were incubated on the BHI agar plates to count microorganism colonies and assess the viability.

### Crystal violet assay

The crystal violet assay used in this study was carried out in accordance with a study published previously^39^. Briefly, disks with multispecies biofilms after 48 h were washed twice with PBS and fixed for 15 minutes using 99% methanol. Then, the well contents were aspirated and the plates were allowed to dry. The biofilms were stained with 1 mL 0.1% crystal violet solution for 5 min. Excess stain was gently rinsed off with tap water and the plates were dried thereafter. The obtained stain was resolubilized in 1 mL of 95% ethanol with shaking in an orbital shaker for 30 min, and the ethanol was transferred to a new 96-well-plate. The solution absorbance was then measured using a Thermo Scientific Multiskan GO reader (Thermo Fisher Scientific Inc., Waltham, MA, USA) at 595 nm.

### Biofilm structure detected by scanning electronic microscopy

Specimens for scanning electronic microscopy were prepared as described above. The disks with 48 h biofilms were gently washed twice with 2 mL of PBS and fixed with 2 mL of 2.5% glutaraldehyde overnight. The specimens were rinsed with PBS and then subjected to graded-ethanol (50%, 60%, 70%, 80%, 90%, 95%, and 100%) dehydrations, with 15 minutes in different concentration of ethanol. The specimens were then sputter-coated with gold and examined by scanning electronic microscopy (SEM, Quanta 200, FEI, Hillsboro, OR, USA)^40^. Each group contained 6 samples and each biofilm was scanned in at least five randomly selected positions.

### Live/dead bacteria staining

The biofilms after 48 h were washed twice with PBS and stained using the BacLight Live/Dead bacterial viability kit (Molecular Probes, Eugene, OR, USA) following the instructions provided by manufacturer^41,42^. Live bacteria were stained with Syto 9 and dead bacteria were stained with propidium iodide. Biofilm images were detected using a Leica DMIRE2 confocal laser scanning microscope (CLSM) (Leica, Wetzlar, Germany) equipped with a 63× (1.4 numerical aperture) oil immersion objective lens. Each biofilm was scanned in at least five randomly selected positions.

### Fluorescent *in situ* hybridization (FISH)

For FISH analysis of biofilm cells, the species-specific probes and the fabrication of the specimens were carried out according to a method described previously^26^. Briefly, the biofilms were fixed in 4% paraformaldehyde. The fixed disks were then washed with phosphate-buffered saline and stored at 4 °C. Next, the fixed biofilms were permeabilized using lysozyme (30 mg/mL) in 100 mM of Tris-HCl (pH = 7.5) and 50 mM EDTA for 20 minutes at 37 °C. The biofilms were then washed with ultrapure water and dehydrated with 50%, 80% and 96% ethanol for 3 minutes, respectively. The dehydrated disks were then inoculated with 30 mL hybridization buffer containing 20% formamide and the oligonucleotide probes. Each of the probes was then diluted in the hybridization buffer to an approximate concentration of 20 ng/mL. After 90 minutes of hybridization at 46 °C, the hybridization buffer was removed, and the chambers were washed with washing buffer (20 mM Tris-HCl buffer, pH = 7.5, containing 5 mM EDTA, 0.02% sodium dodecyl sulfate, and 220 mM NaCl) for 15 minutes at 48 °C. Then, the chambers were rinsed with ultrapure water and the biofilms were imaged using a confocal laser scanning microscope (Leica, Wetzlar, Germany). Each biofilm was scanned in at least five randomly selected positions.

### DNA isolation and quantitative real-time polymerase chain reaction

The total DNA of biofilms incubated for 48 h was isolated and purified using a Yeast/Bact Kit B (QIAGEN Science, Maryland, USA) following the instructions provided by manufacturer. The purity and concentration of DNA were detected using a NanoDrop 2000 spectrophotometer (Thermo Scientific, Waltham, MA, USA). Quantitative real-time PCR was used to quantify the absolute number of *E. faecalis, S. gordonii, A. naeslundii*, and *L. acidophilus*, performed on a C1000 Touch™ Thermal Cycler instrument (Bio-Rad, Philadelphia, PA, USA) with the SYBR reagent (Takara, Dalian, China). The BLAST tool on NCBI (http://blast.ncbi.nlm.nih.gov/Blast.cgi) was used to compare the genomes and to design species-specific primer. The sequences of the primers for the four bacteria species can be found listed in Table 1.

**Table 1:** Specific Primers used for qPCR.

Bacteria	Sequence (5′- > 3′)	Template strand
*E. faecalis*	F	ATTGGAAAGAGGAGTGGCGG
	R	TGAGCCGTTACCTCACCAAC
*S. gordonii*	F	GAGTGCTAGGTGTTAGGCCC
	R	CCTGGTAAGGTTCTTCGCGT
*A. naeslundii*	F	CTCGACACCGTGAAGTTGGA
	R	CGACTTCGTCCCAATCACCA
*L. acidophilus*	F	TGGGGAACCTGCCCCATAG
	R	GGTAGGCCGTTACCCTACCA

### Statistical analysis

All the experiments were repeated at least 3 times independently. Statistical analysis was performed with the SPSS software, version 16.0 (SPSS Inc., Chicago, IL, USA). One-way analysis of variance and Student-Newman-Keuls test were used for all pairwise comparison. Significant differences were considered when *p* < 0.05.

## Supplementary information

A novel antibacterial resin-based root canal sealer modified by Dimethylaminododecyl Methacrylate

## Data Availability

The datasets generated and analyzed during the current study are available from the corresponding author on reasonable requests.
