# Supplementary material for: A novel antibacterial resin-based root canal sealer modified by Dimethylaminododecyl Methacrylate
Source: Sci Rep. 2019 Jul 23;9:10632. doi: 10.1038/s41598-019-47032-8 (PMC6650501; doi:10.1038/s41598-019-47032-8)
Supplement: Supplementary file 1 — A novel antibacterial resin-based root canal sealer modified by Dimethylaminododecyl Methacrylate [file 41598_2019_47032_MOESM1_ESM.docx]

**A novel antibacterial resin-based root canal sealer modified by Dimethylaminododecyl Methacrylate**

Dan Liu^1,2,4^, Xian Peng^1^, Suping Wang^1,2^, Qi Han^1,3^, Bolei Li^1,2^, Xinxuan Zhou^1^, Biao Ren^1^, Hockin H.K. Xu^5^, Michael D. Weir^5^ , Mingyun Li^1,^*, Xuedong Zhou^1,2,^* , Lei Cheng^1,2,^*

^1.^ State Key Laboratory of Oral Diseases & National Clinical Research Center for Oral Diseases, West China Hospital of Stomatology Sichuan University Chengdu 610041 China

^2.^ Department of Cariology and Endodontics, West China Hospital of Stomatology Sichuan University Chengdu 610041 China

^3.^ Department of Oral Pathology, West China Hospital of Stomatology Sichuan University Chengdu 610041 China

^4.^ Stomatological Hospital Affiliate to Zunyi Medical University Zunyi Medical University Zunyi 563000 China

^5.^Department of Endodontics, Periodontics and Prosthodontics, University of Maryland Dental School, Baltimore, MD21201, USA

For: *Scientific Reports*

Submitted in Jun. 2018

**Appendix**

**Materials and Methods**

**Minimum Inhibitory Concentration**

*Enterococcus faecalis* (ATCC29212), *Streptococcus gordonii* (ATCC35105)*, Actinomyces naeslundii* (ATCC12104), and *Lactobacillus acidophilus* (ATCC4356) provided by State Key Laboratory of Oral Diseases (Sichuan University, Chengdu, China) were cultured in brain-heart infusion Broth (BHI; Difco, Sparks, MD, USA) at 37 °C anaerobically (90% N_2_, 5% CO_2_, 5% H_2_). The minimum inhibitory concentration of DMADDM to these four bacteria was tested via serial microdilution assays, respectively, following a previous study[^1^](#_ENREF_1). Briefly, DMADDM monomer was dissolved in BHI broth to a concentration of 16 mg/mL. From this starting solution, serial two fold dilutions were made into 1ml volumes of BHI. Overnight cultures of each bacteria was adjusted to 1x10^6^ colony-forming units (CFU)/mL with BHI broth, and 50uL of inoculum was added to each well of a 96-well plate containing 50uL of a series of antibacterial monomer dilution broths. BHI with 1x10^6^ CFU/mL bacteria suspension without antibacterial agent served as negative control. The wells were read for turbidity after 48 h of anaerobic culture, referenced by the negative and positive control wells. MIC was defined as the endpoint where no turbidity could be detected with respect to the controls.

**Scanning Electron Microscopy for Blank Samples**

The specimens for the biofilm experiments were prepared following a study described previously in the literature [^2^](#_ENREF_2)^,^[^3^](#_ENREF_3). Briefly, composite disks were fabricated using the cover of a sterile 48-well plate as a mold. 20 mg of the sealers containing DMADDM or the control sealer were applied on the surface of each composite disk and flatted using a spatula. The specimens were then placed into a 24-well plate and incubated at 37 °C with 100% humidity for 10 days. Next, the samples were sterilized in an ethylene oxide sterilizer. The disks were gently washed twice with 2 mL of PBS and fixed with 2 mL of 2.5% glutaraldehyde overnight. The specimens were rinsed with PBS and then subjected to graded-ethanol (50%, 60%, 70%, 80%, 90%, 95%, and 100%) dehydrations, with 15 minutes in different concentration of ethanol. The specimens were then sputter-coated with gold and examined by scanning electronic microscopy (SEM, Quanta 200, FEI, Hillsboro, OR, USA)[^4^](#_ENREF_4). Each group contained 6 samples and each biofilm was scanned in at least five randomly selected positions.

**Statistical Analysis**

All the experiments repeated at least 3 times independently. Statistical analysis was performed with the SPSS software, version 16.0 (SPSS Inc., Chicago, IL, USA). One-way analysis of variance and Student-Newman-Keuls test were used for all pairwise comparison. Differences were considered significant when *P* < 0.05.

**Appendix References**

1 Cheng, L. *et al.* Dental primer and adhesive containing a new antibacterial quaternary ammonium monomer dimethylaminododecyl methacrylate. *Journal of Dentistry* **41**, 345-355, doi:10.1016/j.jdent.2013.01.004 (2013).

2 Wang, S. *et al.* Antibacterial effect of dental adhesive containing dimethylaminododecyl methacrylate on the development of Streptococcus mutans biofilm. *International journal of molecular sciences* **15**, 12791-12806, doi:10.3390/ijms150712791 (2014).

3 Li, F. *et al.* Effects of a dental adhesive incorporating antibacterial monomer on the growth, adherence and membrane integrity of Streptococcus mutans. *J Dent* **37**, 289-296, doi:10.1016/j.jdent.2008.12.004 (2009).

4 Han, Q. *et al.* Anti-Caries Effects of Dental Adhesives Containing Quaternary Ammonium Methacrylates with Different Chain Lengths. *Materials (Basel)* **10**, doi:10.3390/ma10060643 (2017).

Appendix Table 1. Minimum Inhibitory Concentration (MIC) of DMADDM on oral bacteria.

| Bacteria | MIC (μg/mL) |
| --- | --- |
| *E. faecalis* | 15.625-31.25 |
| *S. gordonii* | 15.625-31.25 |
| *A. naeslundii* | 31.25-62.5 |
| *L. acidophilus* | 7.8125-15.625 |


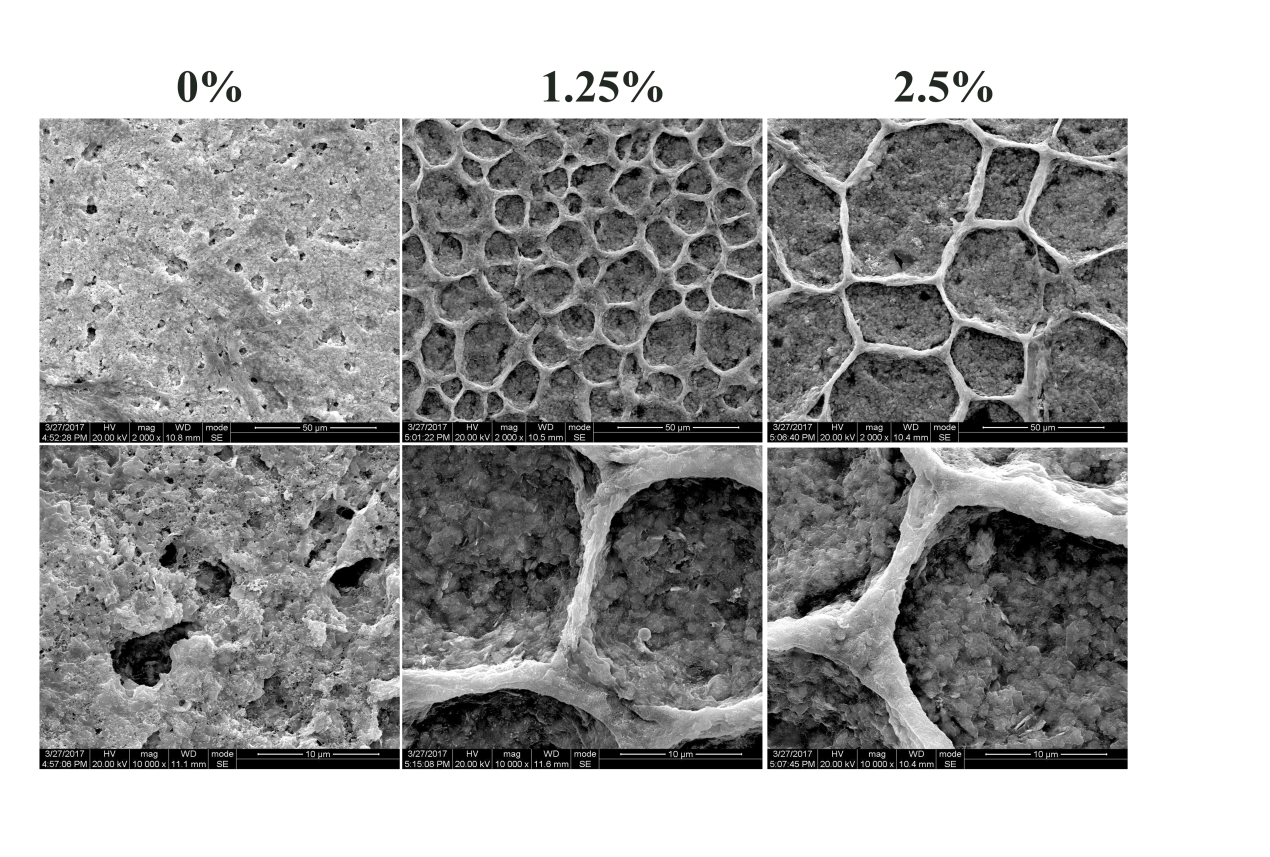


Appendix Figure 1. The scanning electron microscopy images of the blank samples, sealers containing different mass fracture of DMADDM, without any microorganism on the surface. We can see the structure like grid on the surface of sealers’ containing DMADDM, and absence on the group of 0%, too.
